# Playing with the cultural pilgrimage to stimulate tourism: the xFORMAL project on cultural heritage and informal learning.

**DOI:** 10.12688/openreseurope.15321.3

**Published:** 2024-08-13

**Authors:** Anna Siri, Annalisa Di Nuzzo, Simona Marchesini

**Affiliations:** 1 UNESCO Chair in Anthropology of Health. Biosphere and healing systems., University of Genoa, Genova, Italy; 2 Pegaso University, Naples, Italy; 3 Università Suor Orsola, Napoli, Italy; 4 ALTERITAS – Interazione tra popoli, Verona, Italy

**Keywords:** Cultural Heritage; Cultural Pilgrimage; Tourism; Anthropology; Sociology; Education 4.0; Gamification; Science Education; Citizen Engagement

## Abstract

The focus on culture as an engine of sustainable development has favoured its gradual acquisition by institutions engaged in the protection and promotion of heritage as an enabling and systemic factor capable of connecting innovation, continue education, research, and citizen engagement in a single chain.

Knowledge of the landscape that combines works of nature and humankind and its bio-cultural diversity makes it possible to identify innovative informal education and new tourist itineraries where the real experience is presented as a cultural pilgrimage.

The European project of the Marie Skłodowska-Curie Programme RISE ‘Informal and non-Formal E-Learning for Cultural Heritage - xFORMAL’, currently halfway through its lifecycle, was born to create an informal way of accessing the cultural heritage of one's territory, revealing its past, history, and the civilisations that preceded us.

After two years of closure due to the pandemic, this project reintroduced an authentic experience through gamification, a contact with landscapes, museums, and archaeological sites with physical, social, and cultural dimensions of their environments across space and over time.

Building upon this foundation, the article delves into the intricate design and architectural principles that underpin the creation of the xFORMAL game, showcasing it as a paradigm of informal learning. This exploration includes a detailed analysis of the game's innovative design elements, educational strategies, and its role in facilitating an engaging and immersive learning experience outside traditional educational settings.

## Introduction

As we have seen over the past two years, the crisis generated by Covid-19 has heavily affected the cultural and arts sector, in particular impacting museums, cultural venues, all event-related sectors and physical (venue-based) venues. Following their closure, digital content and other experiments aimed at common goals between innovation, culture, education and digital have proliferated.

Again, intending to mitigate the economic and social effects caused by the pandemic, Europe has also endowed itself with an exceptional temporary financial measure that is at the heart of NextGenerationEU in which culture plays an essential role in restarting under the banner of common priorities, including sustainability, resilience and cohesion.

In the 4.0-dimension of the NextGeneration EU, the third component of the first mission, Digitalization, Innovation, Competitiveness, Culture and Tourism, focuses on tourism and culture. In this context, a specific measure devoted to cultural heritage for the next generation has been outlined, with investments aimed at fostering the creation of a digital heritage of culture, improving physical, sense-perceptual, cultural and cognitive accessibility through effective training actions.

Heritage education plays a relevant role, in its plural and interconnected dimension with territories and all citizens and in particular with old and new generations, in the global spirit of the 2030 Agenda that recognises all cultures and civilisations as 'crucial factors for sustainable development'. Due to its cross-cutting role concerning the environmental, social and economic dimensions of sustainable development, culture is a vector for strengthening unity and inclusion and a powerful driver of growth towards improving and promoting the quality of lifestyles.

This framework is fully in line with UNESCO, which in 2019 published the Thematic Indicators for Culture in the 2030 Agenda (Culture|2030), a set of 22 thematic indicators such as quantitative, economic and non-economic, to measure and monitor the contribution of culture itself to sustainability. The framework highlights its 'transformative' role, and that of the related organisations, both as a factor (driver) and as an agent (enabler) of change for the realisation of new imaginaries, a facilitator of inclusive processes, skills and systems for the completion of dedicated programmes.

Culture is therefore placed in a position of proactive confrontation aimed at regenerating forms of active and aware citizenship from a perspective of cooperation between countries.

As a confirmation of the sensitivity of the topic, the first Group of Twenty (G20) on Culture held a webinar on 'PRIORITY 3, Building Capacity through Training and Education. Human Capital. The Driver of Culture-led Regeneration’ to help stabilise the role of culture itself within the G20 Agenda ^1^. The need to promote educational and training actions on culture- and heritage-related values emerges as a condition for the future sustainability of the cultural and creative sectors and is one of the foci of the *Rome Declaration of the G20 Culture Ministers* ^2^.

The document -intended to enshrine the commitment of the parties involved to place culture among the significant economic nodes of the planet's future, integrating it into political and economic planning across the different agendas- reaffirms the value attributed to capacity-building through training and education,

*highlighting the role of education and interpretation of cultural heritage to promote appreciation and respect for cultural diversity' as well as the construction of the meanings of heritage itself as a tool to facilitate ‘the conservation, safeguarding and transmission of shared values and know-how to future generations ^2^.*

Europe is renowned for its exceptional skills in the field of cultural heritage. But European excellence in heritage preservation and conservation is jeopardised by the combined effect of the age pyramid and cuts in public budgets; this affects the transmission of heritage knowledge and skills to younger generations.

The adoption of the *New European Agenda for Culture* ^3^ is at the heart of a relevant series of initiatives promoted by the European Commission (EC) in support of cultural potential. According to the Agenda for Culture,

*Europe's rich cultural heritage and dynamic cultural and creative sectors strengthen European identity, creating a sense of belonging. Culture promotes active citizenship, common values, inclusion and intercultural dialogue within Europe and across the globe. It brings people together, including newly arrived refugees and other migrants, and helps us feel part of communities. Culture and creative industries also have the power to improve lives, transform communities, generate jobs and growth, and create spillover effects in other economic sectors.*

It represents a significant step with a view to the recognition of a common identity, the development of learning mobility and the construction of a European space for education by 2025.

Focusing on culture, with an approach to diversity and collaborative relations as cohesive factors, the New Agenda aims, among other things, to promote art and culture itself in education to implement collaborative arrangements capable of involving territories in forms of enhancement capable of activating light, sustainable and attractive economies (Council conclusions on the Work Plan for Culture 2019–2022) ^4^.

In May 2019, the European Framework for Action on Cultural Heritage ^5^ was published by the European Commission, the first document to recognise the quality of the spil-lover effects of a shared cultural heritage on European societies. This framework identifies five key pillars through which to connect heritage to places and communities: improving public access and involvement through digital means; increasing social and economic capital and environmental sustainability; combating illicit trafficking of cultural heritage, increasing the quality of physical interventions on heritage and protecting against natural disasters and climate change; using technologies for innovation on heritage for the benefit of social innovation and capacity building in the sector; and increasing international cooperation.

Furthermore, the final approval on 23 September 2020 of the Council of Europe's Framework Convention on the Value of Cultural Heritage for Society, signed in Faro in 2005, has put cultural policies on the track of the new approach to heritage, promoting a broader understanding of cultural heritage and consequently opening a new phase for educational design ^6^.

In the current scenario, cultural heritage constitutes a tool rather than a shared space in which to act for the cultural, social, economic and environmental development of a territory and within which to comply with the sustainability principles identified by the seventeen strategic goals (SDGs) of the 2030 Agenda ^7^.

The right to inclusive and equitable quality education and the promotion of lifelong learning opportunities for all are themes in which the contribution of education to (and through) cultural heritage plays a not insignificant role ^8, 9^. In this framework, it is essential to consider cultural diversity as a factor of human development and heritage, in line with the *UN Convention on the Rights of Persons with Disabilities*. The principles recalled in it, like the Article 30, are dedicated to Participation in cultural and recreational life, leisure and sport, are still of current reference for every national and international document on the subject, such as, for example, at the EU level, the recent *Strategy for the Rights of Persons with Disabilities for the period 2021- 2030* ^10^, adopted by the European Commission on 3 March 2021.

The change of perspective for the training of the individual has given the acquisition of competencies, also in informal and non-formal learning contexts, a substantial value for personal growth, approach to society and in the world of work ( *Recommendation of the Council of the European Union on key competences for lifelong learning of 22 May 2018*, new version (2018/C 189/01) updating the previous one (2006/962/EC) ^11^.

The functional specificity in open and virtual access learning environments, moreover, makes heritage education sustainable; keeping the implementation of available modalities to guarantee criteria of substantial equality for the dissemination of knowledge and continuous training possible and repeatable over time and conveys, together with respect and responsibility towards heritage, principles of protection, valorisation, and citizenship.

## xFORMAL project

The European project of the Marie Skłodowska Curie Programme – Research and Innovation Staff Exchange entitled " xFORMAL-Informal and Non-Formal E-Learning for Cultural Heritage" acts in the framework in the previous paragraph illustrated.

xFORMAL aim at creating an informal way of accessing the cultural heritage of one's territory, revealing its past, its history, the civilisations that preceded us, and the common value.

The project will realise this goal through an interactive real and virtual experience that alternatively combines the real world, i.e., a cultural pilgrimage in urban/suburban spaces selected by the xFORMAL Consortium, and an online game where players will find clues to unravel, puzzles to solve, and challenges to embrace.

Devoted to students, educators, families, and the larger community, xFORMAL game intersects the head, heart and hands; the 'three H'. More specifically, its goal is to educate the ‘head’, promoting authentic understandings of European multifaceted cultural heritage (Heritage Education); engage the ‘heart’ of people, encouraging a sense of commitment to Europe, and a sense of responsibility to the environment and the global society (Citizenship Education, Sustainable Tourism); and empower the ‘hands’, integrating technology in education for active participation in the betterment of society (Education 4.0).

In addition to awakening the *Key Competences for Life Long Learning*, xFORMAL awakens interest in history, perceived not as something distant and mediated by formal learning but as something with which to have a direct and immediate relationship. Its soul is based on a real experience presented as a cultural pilgrimage, and the narratives developed enable reconstructed cultural scenarios of the ancient world before Romanisation in Italy, France, Spain, and Poland.

With the help of a smartphone game application, people encounter the elements of this landscape, gets to know them, studies them, and plays with them. On that basis, the xFORMAL game is an informal learning environment with an educational potential involving interdisciplinarity and the acquisition of knowledge and digital, social and cognitive skills.

The xFORMAL project fits entirely into international framework since it is:

- Inclusive: citizens and communities will be included in the experience of their landscape and cultural heritage.

- Participatory: citizens will be invited to play an active role in the process of acquisition of the cultural landscape, participating in the planning, managing and protecting their heritage.

- Building capacity of involved actors: all the stakeholders will have equal ‘voice’ in the Participatory Action Research promoted by xFORMAL.

- Sustainable: a bottom-up approach will create direct benefit to the communities, by strengthening the relationships among them to foster local ownership and shared responsibility.

## xFORMAL theoretical framework

People today need a broader and deeper set of skills to work, communicate, access information, products and services, and participate in social and civic life. Citizenship education and heritage education share common principles and goals to enhance participation, engagement, and cultural awareness. They are extraordinary tools which can be harnessed to help us understand change and continuity in objects, ideas, traditions, and everyday life. We can foster people's critical awareness to reinterpret their culture through heritage education. In doing this, we ensure processes of social empowerment that enable us to competently preserve a commonly shared past and our cultural heritage as European citizens. The challenge, therefore, is to create channels of communication between the main actors involved in the management of heritage and, at the same time, to promote the engagement of society as the primary recipient and legatee of heritage itself.

With all this in mind, heritage education turns out to be one of the main dimensions of citizenship education. It plays a key role in training critical citizens, in the promotion of intercultural education and in the construction of local identities equipped to face the challenges of global citizenship. However, educating with, from, towards and for heritage implies a revision of learning contents and pedagogical tools.

From a lifelong learning perspective, youth and adults should possess skills predicted by rapidly changing technology; they should be conducted but not instructed; they should have guided accessibility to information.

Education 4.0 is a focused approach to learning that aligns with the fourth industrial revolution and is about transforming the future of education using advanced technology and artificial intelligence. Personalised learning, available anywhere, anytime, collaborative and engagement tools allow for expanding skills required to live in our changing society.

Creativity is the foundation of Education 4.0, which eschews theoretical knowledge and pushes learners to learn time management along with the organisational, and collaborative skills essential for the road ahead.

The progressive shift from informal learning and entertainment to online platforms and the virtual dimension is likely to negatively affect the perception of cultural landscape and heritage and contribute to a progressive loss of contact with history and its documents on the ground.

It follows that starting from the educational level, we need to steer society towards a selection of effective strategies to enable citizens to appropriate their own and others' landscapes through direct exploration, conveyed by refined and reasoned methodologies made available from technology, as suggested by xFORMAL on the basis of the theoretical framework described hereafter.

### Heritage education

Transversal to knowledge, languages and cultures, heritage education today finds itself having to contribute to the new formative and inclusive challenges and to strengthen, in a dynamic and flexible sense, both the experimentation of an innovative system for knowledge and skills-transfer and an open management system in the sign of sustainability and cultural welfare. In particular, the change of perspective on the centrality of people and communities, in line with the Faro Convention, requires increasingly advanced interactive forms of planning and various collaborative relationships oriented to include territories, schools and universities. In recognising tangible and intangible heritage as a widespread and ever-changing resource, citizens and communities realise its value as a 'common good', as suggested by the *Strategic framework for the EU's cultural policy*.

In this framework, the *European quality principles for EU-funded interventions with potential impact upon cultural heritage* (ICOMOS 2019) guidelines that were drafted are based on the principle of cultural heritage being a 'common good'.

The contribution of 'heritage communities' to the cultural domain opens up the construction of meanings and content capable of generating new culture, the joint assumption of civic responsibilities and shared management choices between institutions and local actors, thus making the recognition and sharing of social memory indispensable ^12^. Memory is not a property of intelligence, but the basis, whatever it may be, on which concatenations of actions are recorded. According to Zagato ^12^, memory, long thought to be a typical individual faculty, has a profoundly social and collective nature: our memories are shared by many other people in our group or our generation so that the memory of the individual is always interwoven with his or her affiliations. There is no identity of the individual nor a collectivity that does not rest on memory understood both as an interpretation of the past and as a prospect towards a future reality, desired or feared. It is in the social context that people, as a rule, acquire their memory. It is always in society that they recall, recognise, and locate their memories.

Collective memory contains an undeniable individual dimension. The past never remains as it is, but is constantly selected, filtered, and restructured in the terms posed by the demands and needs of the present at both an individual and societal level. It is illusory to believe that our memories must remain unchanged over time and that, if forgotten, it is only a matter of rediscovering their primitive imprint or, at most, correcting the deformations they have undergone. Instead, we must think of memory as an active, living force.

As a symbolic system, collective memory is part of the culture. According to Halbwachs ^13^, memory plays an important role in defining the cultural system understood as 'a series of conceptions inherited and expressed in symbolic form using which people communicate, perpetuate, and develop their knowledge about life and their attitudes towards it'. The collective memory of a group is, for Halbwachs, a set of representations of the past that are preserved and transmitted among its members through their interaction. A synthesis of remembered events and notions, it is also a shared way of interpreting them. Anecdotes, tales, life stories, proverbs, catchphrases, instructions for practical life, common sayings and symbols become syntheses of elements that emerge in interaction and impose themselves on each one as a somewhat codified resource. Within this framework, their stories take on a narrative form and their actions an order taken for granted insofar as they refer to shared and handed-down norms, values, and symbols.

As the foundation of identity, collective memory is core in the representations concerning the group's (historical and mythical) origins. It recalls and reinforces the values and norms intrinsically linked to the group's cultural heritage. As Halbwachs demonstrated, each group incessantly selects and reorganises images of the past with the interests and projects that predominate in the present. This process is intrinsically related to the places and the transformations of those places that the community and the relationships it weaves with the opportunities that gradually present themselves. Symbolic aspects, rituals, and land use make possible what is called the ethnicization of the landscape ^13^.

A place of memory, therefore, needs precise historical and scientific organisation work: the choice of the place, the collection of documentation, the approval of the place by the group or the community, the setting up of a material (museums, house-museum, monuments) or virtual (internet) itinerary, the duration of the place, a valid textual structure that allows the traces of memory present in that particular place to be read and shared by the social group. The specific experiential tourism that is being implemented of material memory places, their sense of stability, given by the real presence of certain traces, guarantees the collective memory a dimension of continuity and favours what Eviatar Zerubavel defines as ‘highly reassuring conservative illusion that nothing fundamental has really changed’ ^14^.

The phenomena of ‘globalisation’, ‘democratisation’, ‘massification’, and ‘mediatisation’, which have characterised Western society over the last half-century, have also invested, on a social and cultural level, the practices of memory. Memory and history are no longer a unicum oriented towards constructing a past, an identity, and a memory common to the nation. Within this perspective of change, every community must write its history, and every social group must do the same to define and continuously redefine its cultural identity, especially in anthropological terms. This becomes imperative for any social reality, from community to association, from group to ethnic group, down to the individual.

The tradition, of which memory is the bearer, is not something mechanically transmitted and passively learned: it can be creatively received, modified and rewritten to the point of being authentic. 

The place of memory, as a synthesis of one's belongings, thus also becomes a counter-memorial place that inspires new perspectives against the tyranny of the dominant historicized memory ^15^. On the other hand, the danger inherent in this reappropriation of places and cultural heritage is that of invented memory of the past as a way of creating a new sense of identity for ruler and ruled ^16^.

Cultural assets are understood as material and immaterial heritage and represent fundamental elements of a community's experiences. Reconstructing and re-appropriating the roots of specific collective identity without losing sight of the global location of cultures is today a strong point of the new glocal dynamics (e.g., having features that are both local and global) in a genuinely transcultural sense. With a view to the valorisation, fruition and patrimonialisation of the territory, space must also be recognised as the cultural form of the overall physical space (physical-environmental-symbolic territorial) and, therefore, the 'subject' of the forms of the landscape and beyond. The traces of human action remain imprinted on things and people and constitute that extra element that makes the construction of a community unique.

The Parish Maps, for example, used by the NGO Common Ground, founded by Sue Clifford in 1983, constitute for anthropology a sort of reconstruction/re-appropriation of the community subjects both as bearers of culture and as builders of memory, to realise the definition of a ‘place of beauty, wonder, liveability’ not only of space. Through maps, the young generations, appropriately guided, can be the glue between old and new for pleasurable heritage tourism that induces an emic self-awareness and an identification of one's resources.

In an integrated system relating to cultural, natural and economic assets as a whole, it is necessary to promote projects for the valorisation, preservation and enjoyment of the landscape, historical heritage and popular tradition around which the inhabitants themselves, even then perhaps in a network with other neighbouring municipalities, can rediscover their roots, their identity, their ability to create, strengthen and enhance the image of the territory, also through the revitalisation of tradition. 

The sense of a Community Map, as delineated by the most innovative strands of anthropological research borrowing from the English experience of Parish Maps, consists in the concrete possibility of reconstructing and defining, in a continuous and vital process, the way through which the community represents itself to the world ^17^. On this line, the European Landscape Convention also expresses recognition that effective territory protection can only be implemented with effective social involvement.

Community maps, then, are the starting point for making cultural heritage a resource that can concretely serve everyone and the entire development process, to address the economic, social and environmental challenges of globalisation. The Community map represents the real re-appropriation by the community of a symbolic yet concrete experienced space that can act as a positive driving force for a definitive and overwhelming take-off of the area.

One tool that can facilitate this process and that appears more suitable than others is the Ecomuseum with the related construction of experience tourism routes.

The Ecomuseum encompasses a plurality of assets that are also very different from each other, such as oral traditions, festivals and rituals, techniques, and knowledge, and so on ^18, 19^.

The objective of Ecomuseums – emphasises Antonia Bertocchi

*is the enhancement of links with the territory, understood both as a more or less intact natural environment and as a man-made environment, i.e., modified by the presence of man and by the type of environmental impact caused by human activities and work ^20^.*

In this sense, the cultural landscape can and must be incorporated as an efficient resource in the strategies of what anthropologists, particularly in the South American area, call ‘Ethno-Development’, since it is one of the components of what we can identify as a community's 'own resources' ^21, 22^.

Therefore, the enhancement of the cultural landscape must not be reduced, as most of the proposed plans and interventions do, to a container of culture, but its role as a generator of culture must be recognised when it functions as a reference of identification for those who perceive it as the indissoluble heritage of the social group.

Undoubtedly the path is long but, in the short term, small constant changes can be promoted. The collection, selection, and cataloguing of the tangible and intangible culture of the community can be realised with structured meetings in which citizens become protagonists to play a multifunctional role. Citizens can become collectors, cataloguers, and users of the heritage of anthropological assets. Still, also, they can be trained in the new professionalism of the sector linked to the knowledge and traditions of the territory, artisan knowledge, popular knowledge and a heritage of oral transmission that could not only be catalogued and collected but also made spectacular by allowing an interactive use of museums.

And in this microcosm of belonging, the relationship between social sciences, art and history becomes ever closer and more fruitful, deeply permeating the theoretical framework of the xFORMAL project.

### Heritage Tourism and cultural pilgrimage

In light of the new modes of travel, it is necessary to rethink the nature of art cities and heritage tourism, which for Italy and the whole of Europe is a gamble not to be missed. The xFORMAL project can be fully incorporated into heritage tourism, since it considers landscape as a fundamental and integral part of the travel experience ^23, 24^.

Tourism, in its most innovative and sustainable declinations, contributes to the integration of cultures and mutual recognition, creating a concrete possibility of fruitful pollution ^25^.

Tourists re-appropriate and share the memory and the territory in the encounter with the host through what is defined as experience tourism: not only the search for new experiences but the search for a social distinction based on an overall lifestyle ^26^.

The benefit, for the visitor/traveller, is not so much a better understanding of the past, the distant, the different, but a reaffirmation of identity through an understanding of the place one occupies in time and space, 'passing through' the places one encounters.

An encounter with otherness defines the dimension of identity. One of the most complex dynamics of the encounter of contemporary societies is determined by mass tourism. In this sense, the anthropology of complex societies defines tourism as a ‘total social fact’ ^27^ in its specific possibility of identifying the modes of impact and encounter between guests and hosts. The phenomenon is ancient but, at the same time, linked to postmodernity and new forms of using time and space ^28^. The use of time in postmodernity has profoundly changed along with the motivations of tourism or travel understood as leisure. Tourism, no longer linked to the outdated vision of leisure time consumption, becomes a dimension of doing; of building culture as social capital, a resource of a community, a symbolic, institutional construction that allows for the definition of horizons of meaning and memory that arise from the encounter between host and guest communities.

Roger Sue speaks of today's 'dominant time' as time freed from the panoptic of traditional capitalist divisions of labour and no longer experienced as worthless 'leisure', as a 'waste of time' ^29^.

Tourism thus determines decisive changes affecting the mentality and behaviour produced by mobilising local resources, both intellectual and material, in the perspective of new development. Several aspects of the territory and the testimonies of the past that inhabit it are condensed into what is called ‘Heritage Tourism’. The main themes of this type of tourism are the landscape, historical dwellings, archaeological, architectural, artistic and natural heritage sites, regional development, special interest tourism, educational excursions, dramatic arts, cultural tours, monument visits, nature trips, the presence of demological interests and traditions combined with post-modernity. It is understood, due to the complexity of the elements involved, that heritage is not only history or reconstruction of past events.

History deals with facts and, as Pirandello points out, the fact in itself is like an empty sack in that it does not stand unless you fill it with a content or a concept (literally, ‘Ma un fatto è come un sacco, veda, che vuoto non si regge. Perchè si regga, bisogna prima farci entrar dentro la ragione e i sentimenti che lo han determinato.') ^30^. Historical facts do not exist until the historian creates them, sometimes even falsely assumed to be true ^31^.

While history is thus based on facts, cultural heritage augments available information and provides interpretations according to a logic of surplus of meaning, which in turn creates 'heritage'. Heritage is, therefore, not only history, but is an interpretive and culturally defined act this also with regard to 'natural' heritage.

It is in this sense that the territory of the cultural district must be understood, which is a way of profitably interpreting this cultural capital of development on the one hand and of self-definition on the other, contributing to redesigning the areas of cultural belonging. This specificity is realised in constructing the self-representation concerning the tourist's imaginaries and modes of encounter.

It is necessary to give the traveller-tourist the possibility of being in the original places where the testimonies of the past lose their arid museum-like character and become sensorially perceptible and experientially recognisable in their original context and its practical immediacy and everydayness. In particular, as the EU invites us to do, it is necessary to rethink and activate tourist flows also and, above all, towards less-known territories and heritage.

Throughout Europe, there has been a significant transformation of experiential tourism, and the anthropology of tourism has contributed to innovative projects since the last decades of the last century. The focus has been on the construction of self-representation, the community of belonging in relation to the tourist's imaginaries. There are many definitions that anthropologists have given to this process: Mc Cannel speaks of the sacralisation of the site ^32^, Butler of tourist space ^33^, Cohen of construction of authenticity ^34^, Graburn of ritual space ^35^, Simonicca of vernacularity of the site ^36^. The constituent elements of this articulated process have to do with the definition of spaces, of places for the enjoyment of tradition, and with the construction of an identity-culture that, as S. Behabib says, is often ‘fictitiously superimposed’ ^37^.

Tourism relates to all aspects of experiences that invest both new forms of holism and continuous reshaping. According to Cohen ^34^ and Mc Cannel ^32^, to name a few of the most accredited theorists, the phenomenon – in addition to economic processes and new planning based on sustainable development – determines a strong cultural change. For S. Zukin, especially in Europe, art towns encourage tourism linked to the deep roots of local history and tradition ^38^. Still, these same motivations also drive visitors to small towns to rediscover lost authenticity and forgotten everyday experiences ^22^.

Special attention is to be paid at an innovative tourism form of tourism, the pilgrimage. Pilgrimages have been undertaken by humankind in all periods of history ^39^. Conceived as a form of communication in space and time by D. Le Bréton ^40^, it is defined as a ‘culture in motion’ by S. Coleman and J. Eade ^41^. But, most importantly, in pilgrimage, the place-centred approach replaces the person-centred approach typical of private worshipping. The contact with the monument– the experience of visiting a museum and working with a piece of stone cut centuries ago– allows citizens to step outside their homes and project themselves towards the outside world, and in this case, towards the historical identity of their urban or extra-urban landscape that belongs to the entire community. Not surprisingly, some scholars have established parallels between pilgrimage and tourism ^42, 43^.

Bauman defines the pilgrim (secular or religious) as a ‘restless seeker for identity’. He assumes that the destination, the set purpose of life's pilgrimage, gives form to the formless, makes a whole out of the fragmentary, and lends continuity to the episodic. Bauman aims to trace the move from pilgrim to tourist, from modernity to postmodernity, since if the modern problem of identity has been to construct an identity and to keep it stable, the postmodern challenge is how to avoid fixation and thus keep one’s options open ^44^. An example is provided by the project 'Verona Minor Hierusalem', where the pilgrimage pathways developed in the town during the Middle Ages – conceived as alternatives to the problematic and perilous routes in the Holy Land – have been revitalised and proposed to citizenships to allow the (re)discovery of parts of the town rich in cultural heritage, usually neglected by tourists and citizens. In a couple of years, these districts have enjoyed new popularity in tourism routes, with clear gains also for the hospitality and reception infrastructures in the city.

Thus, pilgrimage sites are not strictly confined to religious contexts ^45^. 'Post-secular tourism (re)constructs pilgrimage places in novel ways, transcending categorization as modern/secular or traditional/sacred' ^46^.

Consequently, contemporary pilgrimage destinations, such as Ground Zero (the site of the 2001 terror attacks in New York City), Chernobyl (the site of the 1986 nuclear accident in Ukraine), and iconic residences like Elvis Presley's Graceland, are drawing individuals who self-identify as pilgrims ^47^. New Age pilgrimage serves as an intriguing example, representing those who prioritize experiential encounters and inner spirituality. These pilgrimages are characterized as a journey that is ‘rich with meaning’ ^48^, a 'value-laden journey’ ^49^, and a ‘personally meaningful experience’ ^50^.

In summary, the conventional dichotomies that once separated tourists and pilgrims into distinct categories are no longer applicable in the current landscape ^51^.

### Gamification in/for tourism and cultural heritage

In the field of cultural heritage, gamification is a tool used for a variety of purposes, from marketing for tourism purposes, to the protection of intangible and digital heritage assets, to cases where participatory methods are used to address conflicting or difficult aspects of heritage ^52^.

The tourism sector is indeed an industry driven by information and communication technologies (ICTs), which are arguably the strongest drivers of change in tourism ^53– 55^.

Emerging technologies and social media provide interactive platforms for gamification applications ^56, 57^, such as virtual destination tours, blog writing, geo-reference-based games, storytelling, treasure hunts, virtual parks, and can be effectively used in three phases of the journey: before, during and after the trip ^58^. Augmented reality, virtual reality and other emerging technologies have offered various gamification elements to engage tourists more and enhance their experiences ^59, 60^.

Gamification, as a process that utilises elements of game-like thinking, game mechanics, game design and related methods, incentivises user interaction. By using a game-like dynamic, people are encouraged to engage more interactively with an application or system through reward-based feedback. There are several definitions of this concept in the literature, as 61 highlighted in a recent systematic review of the studies and research developed over the years, particularly in five clusters formed when the terms ‘tourism’ and ‘gamification’ were bibliographically linked: Gamification experience for customers, Gamification mechanics and design, Gamification in tourism, Gamification activities and tourist attraction, and Gamification and sustainable tourism.

To summarise, gamification improves website usability ^62^, enhances customer experience and engagement ^63– 65^, increases customer satisfaction ^66^ and helps to strengthen customer loyalty ^67^. Last but not least, it promotes the co-creation of experiences through the active engagement of citizens and tourists, service providers and platforms ^68, 69^.

As a multifaceted tool, gamification has also proven its effectiveness in the protection of intangible and digital cultural heritage assets, providing an innovative means of preserving and presenting valuable cultural elements in an accessible and engaging format.

Finally, a particularly interesting aspect is the use of gamification to address dissonant or difficult aspects of cultural heritage. In contexts where history or cultural elements can lead to tensions or problems of interpretation, the participatory methods of gamification provide a neutral ground for exploring such issues. This approach not only encourages active participation, but also paves the way for a deeper and broader understanding of cultural heritage by stimulating reflection and dialogue ^70^. Ultimately, the integration of gamification in the field of cultural heritage not only enriches the visitor experience, but also helps to preserve and communicate the richness of a territory's history and culture in an innovative way.

### Education 4.0 and Informal education

Pedagogical approaches are reorienting their paradigms toward innovating educational processes to meet the needs of an ever-changing technological society. Knowledge generation in Education 4.0 transcends pedagogy and andragogy to promote an heutagogical approach, a self-learning centred on the learner that encourages self-reflection and metacognition ^71^.

Education 4.0, considered a disruptive educational innovation, has some key features: on the one hand, it proposes a strong integration and fusion of various digital and mobile technologies ^72^ and the availability of open learning environments and educational resources (OER), on the other hand it offers open access, lifelong, individualised and autonomous learning ^73^. Education 4.0, as Fisk ^74^ pointed out, is capable of disaggregating the higher education system in favour of personalised and flexible learning offers ^75^.

In this sense, the heutagogical approach has been suggested as a theory for applying to emerging technologies in distance education and guiding the ways in which distance educators develop instruction using newer technologies such as social media ^76^.

The aim is to ensure that future workers are highly trained in the use of emerging technologies and at the same time the development of interdisciplinary skills that can stimulate reflective thinking ^77, 78^.

On this point, a key role is played by adaptive learning systems ^79, 80^ in which technology is used to promote learning according to the profile and needs of each student ^73^. Education 4.0 takes place in complex virtual learning environments (VLEs), where there is an increased need for interactive and collaborative educational components ^81^.

The integration of smart learning environments into the educational ecosystem has been widely mentioned in the literature, defining the characteristics that a smart learning environment must have ^82, 83^.

Universities, educational institutions and research centres support and encourage educational innovation initiatives and projects to develop new practices involving the application of new technologies.

Gamification, for example, derives popularity from games and their inherent abilities to enhance learning through motivation to action, adoption of engaging game mechanics and dynamics, and problem solving in the most diverse fields of knowledge and life of individuals ^84^.

It is based on the use of elements traditionally found in games, such as storytelling, feedback, reward system, conflict, cooperation, competition, clear goals and rules, interaction, interactivity, among others, in other activities that are not directly associated with games ^85– 87^.

Serious games have been successfully used in many contexts for developing future skills along with the 21st-century learning ecosystem ^88– 90^, as they offer significant advantages, such as combining high-quality content, showing high engagement, and turning mistakes into learning elements ^91, 92^.

In the literature on the subject, it can be seen that the scope of experimentation is mostly within school and university curricula, i.e., within formal education or non-formal education as defined by the Council of Europe.

There is a lack of information, design methodologies and evaluation mechanisms that enable educators to use emerging technologies and pedagogical approaches to provide the right innovative solutions in the field of informal education. According to the Council of Europe, informal education refers to

*a lifelong learning process, whereby each individual acquires attitudes, values, skills and knowledge from the educational influences and resources in his or her own environment and from daily experience. People learn from family and neighbours, in the marketplace, at the library, at art exhibitions, at work and through playing, reading and sports activities. The mass media are a very important medium for informal education, for instance through plays and film, music and songs, televised debates and documentaries. Learning in this way is often unplanned and unstructured ^93^.*

It is estimated that informal learning accounts for about 80% of our knowledge. With the vast amount of data available to everyone in an accessible and constant way, as our information world or 'infosphere' ^94^ looms, the school of the future will be increasingly geared towards supporting the cognitive structure and critical apparatus of the learner able to select data and make the best use of it for their education and growth. However, since a significant part of knowledge will continue to come from informal learning, we have the opportunity to take up this challenge and, as academia and as educators, to convey through this mode, albeit hybrid, the fundamental elements of knowledge in a methodologically sound and effective manner.

## xFORMAL case study

The xFORMAL project is an example of a modern approach to cultural tourism that seamlessly blends informal education with engaging real-world experiences. By combining cultural exploration with elements of gamification and digital technology, xFORMAL transforms traditional tourism into an immersive and interactive journey.

At the centre of this approach is gamification, which serves as a catalyst for engagement. The introduction of interactive elements such as quests, challenges and rewards encourages tourists to actively participate in their experiences. This not only makes the journey more exciting, but also fosters a deeper understanding and appreciation of the cultural heritage they are exploring.

In this context, tourism is transcending its conventional boundaries and evolving into a powerful form of informal education. As tourists navigate historic sites and interact with local art, architecture and customs, they gain invaluable insights. These real-life encounters provide a rich, contextualised backdrop for learning and make the experience both educational and entertaining.

Interactive learning is another important aspect of this approach. Participants are not just observers of cultural heritage, but active participants. By solving riddles related to local history, overcoming challenges that provide insights into the local way of life and participating in guided cultural activities, they experience hands-on and participatory learning. This method enhances the educational value of their trip and makes it memorable and impactful.

Community involvement is an essential part of this dynamic. By encouraging respectful and meaningful interaction with local communities, gamification fosters a sense of shared learning and community spirit. This not only enriches the tourists' experience with real cultural insights, but also contributes to the local economy and cultural preservation.

The integration of digital platforms and mobile apps adds another layer to this experience. These technologies provide additional context and information that complements real-world exploration and enhances both the educational and entertainment aspects of tourism.

In essence, through its innovative approach, the xFORMAL project demonstrates how the combination of cultural pilgrimages and informal learning can revolutionise the tourism landscape. By providing an immersive, educational and entertaining experience, it aims to stimulate interest in cultural heritage and contribute to sustainable tourism development. 

To effectively evaluate the contribution of the XFORMAL project to tourism and education, a combination of methods is planned. Before and after the game, surveys and feedback forms will be available on mobile phones to measure changes in perception and knowledge of cultural heritage, which will help to quantify the educational impact. Data analysis and monitoring is also crucial as digital tools will be utilised to monitor user engagement with the gamification elements of the project. Metrics such as the number of users, duration of sessions and completion rates will provide insight into the attractiveness and educational effectiveness of the project.

In addition, conducting in-depth case studies or interviews with different participants will provide qualitative insights into how the project has influenced their understanding and appreciation of cultural heritage and what impact it has had on social engagement and community dynamics. An economic impact analysis could examine tourism data to assess changes in visitor numbers and economic contributions in project areas and highlight the project's role in promoting sustainable tourism.

Longitudinal studies can be crucial in assessing the lasting impact of the project on local communities, educational institutions and the tourism sector and monitoring changes over several years. Finally, obtaining feedback from cultural and educational experts, together with peer reviews from other professionals in the field, will allow for an external evaluation of the project's methods and results. This comprehensive approach will ensure a robust evaluation of the effectiveness of the XFORMAL project as a model for future projects aimed at linking cultural heritage with education and tourism initiatives.

This approach can serve as a model for future projects that harmonise cultural exploration, learning and social engagement, redefining the nature of cultural tourism.

Moving from the conceptual background based on the framework above, xFORMAL proposes cultural-historical game pathways that allow citizens to rediscover their monuments, museums, and archaeological landscape.

The concept of pilgrimage, to valorise a region or a town's cultural heritage, in xFORMAL project, has been adapted to a period of history, which is usually ignored by the school curriculum, i.e., the pre-Roman cultures. Throughout Europe, the history of this period (which runs from the 8th to the 1st c. BC) is rarely presented in History classes, and citizens ignore what is collected in their museums and landscape related to civilisations before Rome.

Yet, the heritage left by these ancient peoples, such as the Etruscan in Italy, the Gauls in France or the Hibers in Spain is important and relevant for our times: alongside the alphabet, arrived in Europe from Greece during the late 8th c. BCE, mythology, beliefs or technology have their roots well before Greco-Roman civilisation and form a large part of our historical and cultural heritage, which is largely ignored.

### xFORMAL game's ingredients

The xFORMAL project aims to create a context where science and technology meet citizens of all ages in an informal intergenerational educational environment based on a platform and game dedicated to the common European cultural heritage.

The key ingredients of the project are the history of ancient Europe (partly taught in formal education); landscape (typically, a basis for non-formal learning); the heutagogic approach; virtual/augmented reality (generally recognised as a tool for informal learning) and the sharing of knowledge and experience between researchers from the Social Sciences and Humanities and Information and Communication Technologies in a cross-sectoral context.

Intending to contribute to today’s social responsibility of heritage education in its various contexts, xFORMAL produces a continuous interaction with the most current forms of educational approaches. In particular, the project moves from the global one of public archaeology to the conservative one of the conscious memory of the didactics of history and archival sources; from the civil awareness of heritage protection policies, natural and artificial, activated by landscape education to the participative methodologies of the ‘community cataloguing’ of anthropology; from the acquisition of specific skills to the valorisation of creative enterprises; from the opportunities of information sharing and knowledge design to the inclusive design of the latest directions on universal accessibility.

Last but not least, elements such as critical thinking, problem- solving skills, teamwork, communication and negotiation skills, analytical skills, creativity and intercultural competencies are some of the core competencies that the project aimed to intercept.

In the xFORMAL game, obstacles are overcome by moving through a complex world, accumulating adequate tools and booty, until finally, the treasure or goal is reached. This genre focuses on exploration and riddle solving, featuring long-term obstacles without requiring quick reflexes or intense action. The game, that will be available in a release to play on mobile devices at the end of the project, will be developed combining geolocation information with the information retrieved from the platform within a game narrative. The adventure game contains a self-evaluation tool, allows the researchers to answer the main scope of the project, i.e., how informal learning can impact formal education, passing through the intermediation role of Museums and cultural associations.

The game’s mission is to physically explore a pathway and be creative and quick for finishing the game. It is an adventure-packed geolocation game that has several locations to be visited. Each pathway reveals history and ancient landscapes. The location-based game unfolds in a multicultural environment, stimulating players to walk outdoors and explore the surrounding environment. The player is taken through urban or country landscapes, in modern or historical places.

**Aim of the game**

Drawing direct inspiration from the landscapes and cultural heritage selected by the partnership, the xFORMAL game aims to develop skills, competences and knowledge in line with the European Framework of Competences and Skills. This game integrates digital competences through the use of devices such as smartphones and tablets and promotes personal and social competences through museum visits and group or family tourism. In addition, it promotes civic competence through the exploration of public places dedicated to cultural heritage and competence in cultural awareness and expression through the analysis of monuments and historical documents.

The playful part of the game, based on challenges and information gathering, stimulates informal learning by encouraging players to actively participate in the game. From an educational perspective, xFORMAL emphasises the development of soft skills and sees the partnership engaged in researching and developing innovative assessment tools for informal education. The project focuses on a wide range of soft skills, including organisational skills such as time management, critical thinking and strategic planning, as well as collaborative skills such as flexibility, adaptability, collaboration, networking and analytical thinking. It also focuses on the ability to think creatively, social skills such as intercultural skills and personal skills such as personal development, self-management and emotional intelligence.

**The pathway-scenarios**

The game is organised in a framework of ‘pathways’, which will be a sort of pilgrimage into the cultural heritage. In line with the game's aim to promote Europe's oldest identity substrate through experiential on-site activities (visits to museums, archaeological sites) and online cultural games, the consortium has identified scenarios that best represent the linguistic and cultural diversity in the partner countries in the historical period under consideration.

Great attention has been paid to both tangible and intangible cultural heritage, in close connection with the main learning outcomes to be achieved. Participants will learn to contextualise the Palaeo-European cultures geographically and chronologically, identify writing and its use in different cultures and recognise the different types of inscriptions that define a particular epigraphic culture. They will learn about the key characteristics of these peoples and learn about the evidence that helps us better understand these cultures today. By interacting with these cultures, the players will learn how to extract information to gain knowledge. In addition, the xFORMAL game will cover various cultural contacts within the featured areas, including interactions with the Romans upon their arrival. Finally, participants will learn to appreciate the heritage that our history has left us over the centuries.

In the same way as the ancient pilgrims, the gamers will have to gather credentials (tools, awards, points) during the path by solving challenging tasks. xFORMAL called the stations on the pathway with their monuments: ‘scenarios’.

The players move through the landscape on the developed pathways and their devices track their position through geolocalisation. A grid with the reconstructed ancient landscape will always be available on the map so that the continuity between ancient and modern landscapes can be checked.

A short video presents the landscape and the cultural framework in which the players are moving.

Every pathway was designed to be barrier-free, a fundamental principle that reflects the inclusive vision expressed in the United Nations Convention on the Rights of Persons with Disabilities. The process required intensive collaboration with accessibility experts and, above all, with people with disabilities to ensure that each pathway is not only physically accessible, but also intuitive and comfortable for all. A key element is also the feedback from people with disabilities participating in the pilot project (from March 2024) to confirm that the chosen solutions are not only theoretically valid but also effective in practise.

Each pathway consists of several stops (e.g. museum, city, park or archaeological site) where the players find different cultural documents (arch, cippus, stele, inscription, vase, coin, etc.).

The players have to solve several tasks (challenges) for each cultural record (reading letters, measuring the object, recognising the shape, counting, discovering a hidden element, etc). Once all the tasks of each stop are solved, the players will be free to move to the next stop. To solve the challenges, they will have some help (educational kit), which can be unlocked in exchange for certain credentials or gadgets accumulated in previous tasks. 

Each pathway has been designed following an incremental level of difficulty. 

The challenges presented to the player include various activities: filling in blanks with letters, numbers, nouns or strings of graphemes limited to 30 characters including spaces; selecting up to three options in a drop-down menu; selecting specific parts of an image by clicking on them within the game app or environment; and finally, answering true or false to questions based on videos or educational materials provided by the character at each stage of the game. Affording the tasks of the first stops, the players will acquire competencies in solving the following tasks. For example, if at the path's start they recognise the alphabet and some letters written on a monument, in the end, they will be able to transcribe more extended portions of an inscription.

Since xFORMAL is framed historically from the 8th century B.C. to Romanization, it is natural for the player to deal with the Latin language during the game. They will find some Latin inscriptions and Roman monuments and will be accompanied the riddles written in Latin for the duration of the route. The players will have all the possible online resources to understand the language, and they can ask for some help from someone who can understand the Latin language (a parent, a relative, a friend, or a teacher). They will approach another topic as part of the cultural heritage.

Each riddle, taken from collections of Latin texts, such as the most famous *Flumen et piscis*, mentioned in Umberto Eco's novel *The Name of the Rose*, can only be solved by successfully completing all challenges relating to a single monument. For each challenge, players receive a piece of the riddle. Additional tasks, such as counting, calculating or measuring, or tasks relating to inscriptions, can yield prizes in the form of gold rings, bars of strength and pre-Roman or Roman coins. The word collected at the end of each challenge forms the sentence containing the riddle in Latin. Players are rewarded with extra points for solving the riddles and their nickname appears in the overall ranking with all the winners of the game, which is displayed on the project platform.

**Gaming analytics**

xFORMAL adopts gaming analytics to improve its gaming design, according to user preferences, and attract more gamers to play the project game. Data-driven game design effectively engages any gamer at specific levels in the game. The use of game analytics metrics will be used to identify game optimisation points and improve the overall gaming model.

The Game Performance Analysis Dashboard is the heart and soul of the game analysis and is critical for identifying any recurrent problem or elevating the game performance. Thanks to data visualization techniques, dashboards are easily accessible for most users and can be deployed in tracking online player data.

## Conclusion

In recent decades, the cultural offer has multiplied incredibly, thanks to a tourism design that tends to include more and more cultural experiences in its products. There is talk of experiential tourism, participatory tourism, sometimes simply postmodern tourism, if not post-tourism. There are also those who claim that tourism no longer exists and that we are all just city users.

Even the concept of pilgrimage is clearly evolving and blending seamlessly into the fabric of cultural and experiential tourism, which is changing the industry. New pilgrimage centres and concepts are constantly emerging. Evidence of this development is the fact that pilgrimage is taking an increasingly important place in the tourism industry, reflecting the growth of the pilgrimage concept in various tourism segments ^95^. labelled this phenomenon the 'rejuvenation of pilgrimage' and linked it to the search for non-ordinary experiences. He argued that this rejuvenation has resulted in pilgrimage losing its religious element - the supposedly unique identity that distinguishes pilgrimage from other forms of travel.

Regardless of the perspective chosen, the fact remains that the link between culture and tourism has taken on a central importance that was unknown in the era of mass tourism. Culture has become an indispensable resource for tourism innovation, just as tourism has proved to be a possible educational channel for the dissemination of culture itself. However, it would be wrong to consider the multiplication of cultural occasions, as well as the increasingly frequent use of history for edutainment purposes, as the sole result of changes in the way travel and holidays are conceived. New cultural practises are one of the many consequences of the emergence of an ever broader definition of cultural heritage. Memory, intangible heritage, the international or universal dimension are concepts that have transformed the 19th century notion of heritage and opened up new places and practises.

In response to the growing need to revitalise tourism and promote cultural heritage, the xFORMAL project, currently in transition, seamlessly combines real and virtual elements to show how combining a cultural pilgrimage with informal learning can transform the tourism landscape. By providing an immersive, educational and engaging experience, the project aims to stimulate interest in cultural heritage and contribute to the development of sustainable tourism.

This innovative approach can serve as a model for future projects that seek to harmonise cultural exploration, learning and social engagement. The integration of real cultural experiences, tourism, gamification and informal and lifelong education creates a synergistic approach where each element reinforces and complements the others, resulting in an engaging, educational and entertaining experience for participants. Indeed, the xFORMAL project stimulates a coherent approach to cultural tourism, better known as cultural pilgrimage, which transcends conventional boundaries and evolves into a powerful form of informal education.

Through real-life encounters, participants acquire knowledge about local history, art, architecture and customs, enriching their understanding in an authentic context. Gamification acts as a catalyst for engagement and enriches cultural tourism with interactive elements such as quests, challenges and rewards. This strategy motivates tourists to actively participate in their experiences and fosters a dynamic and engaging learning environment. In addition, the integration of digital platforms and mobile applications enhances both the game and the educational aspect.

The virtual game seamlessly transitions into real-world exploration, providing participants with additional context and information that further enriches their overall experience. This interconnected approach ensures that each element contributes harmoniously to an engaging, educational and enjoyable cultural tourism experience. 

The synergy between gamification and cultural tourism leads to interactive learning. Participants not only absorb cultural heritage through observation, but actively engage by solving puzzles, overcoming challenges and participating in guided activities, resulting in a hands-on, participatory learning experience. Community involvement, an important aspect of true cultural and tourism experiences, complements this dynamic. Gamification complements this dynamic by encouraging a respectful approach to the commons, especially those that are less well known and unfortunately less protected and valued, fostering a sense of shared learning and community spirit. 

In summary, at the end of its project cycle, scheduled for August 2025, xFORMAL aims to set a benchmark in the field of cultural exploitation by combining technological innovation and democratised access to cultural heritage, redefining the way people interact with art, history and cultural heritage.

## Ethics and consent

Ethical approval and consent were not required.

## Data Availability

All data underlying the results are available as part of the article and no additional source data are required.
