# Supplementary material for: Playing with the cultural pilgrimage to stimulate tourism: the xFORMAL project on cultural heritage and informal learning
Source: Open Res Eur. 2024 Aug 13;3:93. Originally published 2023 Jun 14. [Version 3] doi: 10.12688/openreseurope.15321.3 (PMC10951561; doi:10.12688/openreseurope.15321.3)
Supplement: Peer review history

# Reviewer report: Reviewer response for version 3

**DOI:** 10.21956/openreseurope.19117.r43102

**Published:** 2024-09-04

**Authors:** Piera Buonincontri

**Affiliations:** 1 Italian National Research Council, Institute of Studies on the Mediterranean, Rome, Italy

I read the revisions by the authors of 'Playing with the cultural pilgrimage to stimulate tourism: the xFORMAL

 project on cultural heritage and informal learning' and in my opinion their changes are now appropriate and sufficient to approve the article without reservations.

Is the case presented with sufficient detail to be useful for teaching or other practitioners?

Yes

Is the work clearly and accurately presented and does it cite the current literature?

Partly

If applicable, is the statistical analysis and its interpretation appropriate?

Not applicable

Are all the source data underlying the results available to ensure full access and reuse by other researchers?

Partly

Are the conclusions drawn adequately supported by the data and analysis?

Yes

Is the background of the case’s history and progression described in sufficient detail?

Partly

Reviewer Expertise:

Tourism experience, smart destinations, cultural tourism

I confirm that I have read this submission and believe that I have an appropriate level of expertise to confirm that it is of an acceptable scientific standard.

# Reviewer report: Reviewer response for version 2

**DOI:** 10.21956/openreseurope.18490.r38142

**Published:** 2024-04-22

**Authors:** Estefanía López-Salas

**Affiliations:** 1 Department of Architectural Design, Urban Planning and Composition, Universidade da Coruna, A Coruña, Galicia, Spain

The authors have taken into account the suggestions made in the previous revision.

 I would just recommend two minor corrections. In page 6 pdf version (second paragraph, third line), there is a repeated sentence (...plays with them, and plays with them). In addition, in page 9 (paragraph 6, first line), where this is written: "In is in this sense...", it should be written "It is in this sense..."

Is the case presented with sufficient detail to be useful for teaching or other practitioners?

Partly

Is the work clearly and accurately presented and does it cite the current literature?

Yes

If applicable, is the statistical analysis and its interpretation appropriate?

Not applicable

Are all the source data underlying the results available to ensure full access and reuse by other researchers?

Partly

Are the conclusions drawn adequately supported by the data and analysis?

Partly

Is the background of the case’s history and progression described in sufficient detail?

Partly

Reviewer Expertise:

Cultural Heritage, Digital Humanities, Monastic Architecture and Landscape, Digital Art and Architectural History

I confirm that I have read this submission and believe that I have an appropriate level of expertise to confirm that it is of an acceptable scientific standard.

# Reviewer report: Reviewer response for version 2

**DOI:** 10.21956/openreseurope.18490.r38140

**Published:** 2024-04-12

**Authors:** Piera Buonincontri

**Affiliations:** 1 Italian National Research Council, Institute of Studies on the Mediterranean, Rome, Italy

The Authors have carefully followed the suggestions by reviewers and I think they have improved their article.

 They have made the link between the XFORMAL project and the theoretical framework at the basis of the project clearer .

 However, I think that they should consider further issues:

 1. The 'challenges typology' section could be deleted, as the same information is provided in 'The pathway scenarios' section.

 2. The 'Latin language for the underlying narrative' section could be deleted, as the same information is provided in 'The pathway scenarios' section.

 3. The 'geographic environment' section could be deleted, as the same information is provided in 'The pathway scenarios' section.

 4. In my opinion, Authors should explain to the readers how they think the effects of the XFORMAL project on tourism and education can be assessed. They write: "By providing an immersive, educational and entertaining experience, it aims to stimulate interest in cultural heritage and contribute to sustainable tourism development. This approach can serve as a model for future projects that harmonise cultural exploration, learning and social engagement, redefining the nature of cultural tourism". It would be interesting if the authors explained how to verify which are the contributions of the project, so to really make it a model for future projects.

 5. There are some minor issues concerning the English language (grammar and punctuations). E.g.: quotation marks in the paragraphs in italics p.4; p. 6 second paragraph (plays with them is repeated); the word 'spillover' at p.5

Is the case presented with sufficient detail to be useful for teaching or other practitioners?

Yes

Is the work clearly and accurately presented and does it cite the current literature?

Partly

If applicable, is the statistical analysis and its interpretation appropriate?

Not applicable

Are all the source data underlying the results available to ensure full access and reuse by other researchers?

Partly

Are the conclusions drawn adequately supported by the data and analysis?

Yes

Is the background of the case’s history and progression described in sufficient detail?

Partly

Reviewer Expertise:

Tourism experience, smart destinations, cultural tourism

I confirm that I have read this submission and believe that I have an appropriate level of expertise to confirm that it is of an acceptable scientific standard, however I have significant reservations, as outlined above.

# Response

**Published:** 2024-05-18

**Authors:** Anna Siri

Dear Reviewer, Thank you for your thorough review and your valuable suggestions. We have carefully reviewed and considered each comment in order to improve our article. In particular, with regard to evaluation, we have added an explanation of how we believe the impact of the XFORMAL project on tourism and education can be assessed. In particular, we have detailed the methods used to assess the project's contributions to cultural heritage and sustainable tourism development in order to make it a model for future projects.

 We hope that these revisions fulfil your expectations and contribute to the overall quality of our article. Thank you once again for your insightful feedback. Best regards, Anna Siri

# Reviewer report: Reviewer response for version 2

**DOI:** 10.21956/openreseurope.18490.r38141

**Published:** 2024-03-19

**Authors:** Chiara Cecalupo

**Affiliations:** 1 Departamento de Humanidades: Historia, Geografía y Arte, Universidad Carlos III de Madrid, Getafe, Community of Madrid, Spain; 2 Classics & Archaeology, Faculty of Arts, University of Malta, Msida, Msida, 2080, Malta

The authors have expanded and improved the problematic parts of the first version of the text. I appreciate their answer to the revievers, the clarity in defining the objectives of the text, the new reflection on the framework (and in particular on gamification) and the deeper explanations concerning the xFORMAL project. I remain of the opinion that a more critical reading on the use of smartphones in the fruition of cultural heritage is necessary, but I believe that the work done by the authors for this second version has corrected the main problems of the original text.

Is the case presented with sufficient detail to be useful for teaching or other practitioners?

Partly

Is the work clearly and accurately presented and does it cite the current literature?

Yes

If applicable, is the statistical analysis and its interpretation appropriate?

Not applicable

Are all the source data underlying the results available to ensure full access and reuse by other researchers?

Partly

Are the conclusions drawn adequately supported by the data and analysis?

Partly

Is the background of the case’s history and progression described in sufficient detail?

Partly

Reviewer Expertise:

Cultural Heritage, Museums, Christian Archaeology

I confirm that I have read this submission and believe that I have an appropriate level of expertise to confirm that it is of an acceptable scientific standard.

# Reviewer report: Reviewer response for version 1

**DOI:** 10.21956/openreseurope.16563.r33572

**Published:** 2023-07-20

**Authors:** Chiara Cecalupo

**Affiliations:** 1 Departamento de Humanidades: Historia, Geografía y Arte, Universidad Carlos III de Madrid, Getafe, Community of Madrid, Spain; 2 Classics & Archaeology, Faculty of Arts, University of Malta, Msida, Msida, 2080, Malta

The article is very interesting and aims to present the fascinating xFORMAL project within a broader theoretical framework. The article has very good potential, and I think it needs to be improved in some points.

 The first three parts offer indeed a broad view of the cultural and political foundations at international level on which the EU-funded xFORMAL project is based. The first section is definitely the most interesting and valuable part of the essay. I really appreciated the comprehensive overview of recent actions made by international bodies for culture and education. It is an excellent summary, very useful for contextualising the project, but which can also be helpful to anyone interested in the topic.

 The bibliography on pilgrimage, tourism and ecomuseums is also correct and appropriate, though brief in relation to the vast number of texts published recently on these topics. However, as this is a work of an international scope, it would be preferable to present broader examples and comparisons. For example, among the reproductions of the Holy Land conceived as alternatives to the routes to Jerusalem, the experience of Verona Minor Hierusalem is very interesting and could be better contextualised by looking at the numerous examples worldwide (see Crispin Paine's most recent bibliography).

 I find that the second part of the article needs revision. First of all, what is presented as an xFORMAL case study does not seem to be a case study, nor is it clear whether it describes a finished work or a proposal. The description of a real scenario would really give strength to the essay.

 In dealing with these themes, however, the authors should better frame the issues of gamification in heritage tourism and learning practices. I suggest looking at further bibliography such as "Tourism Fictions, Simulacra and Virtualities", edited by Maria Gravari-Barbas, Nelson Graburn and Jean-François Staszak, Routledge 2020.

 Similarly, when discussing citizen engagement through digital and virtual, it is important to mention themes of digital tools for heritage (i.e. equality of access to devices, right to disconnection, digital fatigue and digital exclusion). These are key issues, , deeply debated in contemporary museology and heritage use, that cannot be overlooked.

 At last, I have some concerns regarding the use of ‘pilgrimage’ in this case. The word 'pilgrimage' (although always defined in the article as 'cultural pilgrimage') is always strongly characterised by religious aspects. In this text, the concept of pilgrimage is applied to non-religious (and pre-Roman) matters, but this is not always obvious to the reader. I suggest to the authors to clarify very well what they mean by the word ‘pilgrimage’, and to reflect on the fact that this word do not fit in discourses that focus only on the monuments and places, without entering into the central issue of any “pilgrimage”, that is the individual, supernatural perception of the place, as experienced by every single visitor.

Is the case presented with sufficient detail to be useful for teaching or other practitioners?

Partly

Is the work clearly and accurately presented and does it cite the current literature?

Yes

If applicable, is the statistical analysis and its interpretation appropriate?

Not applicable

Are all the source data underlying the results available to ensure full access and reuse by other researchers?

Partly

Are the conclusions drawn adequately supported by the data and analysis?

Partly

Is the background of the case’s history and progression described in sufficient detail?

Partly

Reviewer Expertise:

Cultural Heritage, Museums, Christian Archaeology

I confirm that I have read this submission and believe that I have an appropriate level of expertise to confirm that it is of an acceptable scientific standard, however I have significant reservations, as outlined above.

## References

[1] : Tourism Fictions, Simulacra and Virtualities. *Routledge, Oxford* .2020;

# Reviewer report: Reviewer response for version 1

**DOI:** 10.21956/openreseurope.16563.r33580

**Published:** 2023-07-11

**Authors:** Estefanía López-Salas

**Affiliations:** 1 Department of Architectural Design, Urban Planning and Composition, Universidade da Coruna, A Coruña, Galicia, Spain

The paper presents the project xFormal that aims to test and improve informal or non-formal learning on Cultural Heritage related to the pre-Roman European history, through the use of game-based technologies.

 The paper is well-structured into two main parts, after the introduction. The first one is devoted to the presentation of the xFormal project and its theoretical framework, and the second one is focused on the introduction of the cultural-historical game pathways that xFormal project will developed to promote learning in the pre-Roman cultures.

 The theoretical framework of the project is very well-written, grounded and easy to understand. It is also well-balanced in the presentation of the main research areas related to the study: (1) Heritage education, (2) Heritage Tourism and cultural pilgrimage, and (3) Education 4.0 and Informal Education that, on the whole, offer a brief but really well-explained review of three fundamental areas in which the project is being developed.

 However, the second part of the structure, the case study could be better introduced and discussed in relation to the theoretical framework previously presented. First of all, it is not clear to what extent the case study has already been implemented or if it is a proposal to be developed, currently. In fact, the case study lacks any reference to the real pathway scenarios that have been selected to its development or a discussion about the results in relation the evaluation systems they are said to be implemented. In addition, in this second part of the article there are some repetitions in relation to the information presented in different sections that make the reader wonder if so many sub-sections are needed or if some of them should be joined (i.e. The Pathway-scenarios + Educational kit (already presented at the end of the previous section); Narrative + Unlocking necessary educational tools).

 As in the introduction of the article and in the theoretical framework of the project is given importance to the implication of community in writing its own history, I would recommend the authors to explain how the design of the game, pathways-scenarios, and narratives face that aspect. Does the project propose any co-creative experience in the selection and/or design of scenarios and narratives? If so, how was that process implemented or is it being implemented?

 Another important aspect that both the introduction and theoretical framework point to is universal accessibility, and inclusive design. In fact, it is also point out in the conclusions of the article, but there is a complete lack of information regarding this aspect in the presentation of the case study.

 In my opinion, it is important that the article discusses in the Conclusion how the case study proposed can achieve or has already achieved the challenges presented in the introduction and theoretical framework. This could be faced with a broader discussion in the conclusions, with more clear and direct relations in between the main points of the theoretical framework, the challenges exposed, and the solutions presented in the case study.

 Other points to be considered by the authors are the following:

 1.The first part of the title (Playing with the cultural pilgrimage to stimulate tourism) should be reconsidered as it does not match its content properly. The article presents a particular project which is more focused on testing new forms of informal education on Cultural Heritage through game-based technologies than on stimulating tourism. The article presents the cultural-historical game pathways, the game ingredients and aims in relation to estimated learning competences, so the main focus in this article is mainly on education, but not on a tourist approach.

 2. While the plain language summary is well-written and clear, the abstract could be improved in particular in what respect to the main aim of the article, the case study, to which only a sentence is devoted at the end of the abstract. It could also be improved the way in which the structure of the article is presented and a brief reference to the results.

 3. In the whole text there are some excerpts of external authors that are quote with simple quotation marks and others with double quotation marks. I suggest the authors to use a common way for the quotations. For instance, in the paragraph number 8 of the introduction, we find the following two excerpts: … **"**highlighting the role of education and interpretation of cultural heritage to promote appreciation and respect for cultural diversity **"**… (double quotation marks)

 … **‘**the conservation, safeguarding and transmission of shared values and know-how to future generations **’**. (simple quotation marks)

 This issue should be reviewed in the whole article.

 4. In case of long quotations (more than three lines) such as the ones of the Introduction (Paragraph 10, *Europe’s rich cultural heritage and dynamic cultural and creative sectors…*) and Education 4.0 and Informal education (Paragraph 12, *a lifelong learning process, whereby each…*), I will recommend the authors to include them as a separate paragraph, indented and in italic type, without quotation marks.

 5. There is a minor mistake in paragraph 4 of the Introduction:

*Heritage education plays a relevant role, in its plural and interconnected dimension with territories and all citizens and in particular with old and new generations, in the global spirit of the 2030 Agenda that recognises all cultures and civilisations as **'crucial** factors for sustainable development *[this sentence lacks the closing quotation mark].

 6. There is a minor mistake in paragraph 2 of The pathways-scenarios:

*Each pathway will be composed of multiple stops (e.g., museum, town, archaeological park **o*** [should be **or**] *site) in which the player will find two-four cultural records (arch, cippus, stele, inscription, vase, coin, etc.).*

 7. There is a minor mistake in paragraph 2 of Narrative:

*The Common Room will be a virtual environment. The players will meet and speak to another **via.*** [the point should be removed] *a chat/translation tool embedded in the game.*

Is the case presented with sufficient detail to be useful for teaching or other practitioners?

Partly

Is the work clearly and accurately presented and does it cite the current literature?

Yes

If applicable, is the statistical analysis and its interpretation appropriate?

Not applicable

Are all the source data underlying the results available to ensure full access and reuse by other researchers?

Partly

Are the conclusions drawn adequately supported by the data and analysis?

Partly

Is the background of the case’s history and progression described in sufficient detail?

Partly

Reviewer Expertise:

Cultural Heritage, Digital Humanities, Monastic Architecture and Landscape, Digital Art and Architectural History

I confirm that I have read this submission and believe that I have an appropriate level of expertise to confirm that it is of an acceptable scientific standard, however I have significant reservations, as outlined above.

# Reviewer report: Reviewer response for version 1

**DOI:** 10.21956/openreseurope.16563.r33164

**Published:** 2023-07-03

**Authors:** Piera Buonincontri

**Affiliations:** 1 Italian National Research Council, Institute of Studies on the Mediterranean, Rome, Italy

The article presents a case study of the construction of the game xFORMAL as a technological tool to create an informal way of accessing the cultural heritage. It is an interesting topic and it has been nice to read the study. Despite that, in my opinion, it should be improved for indexing.

 The paper lacks an appropriate theoretical background, since it does not present the main literature review about gamification in tourism, cultural heritage enhancement through technology, and informal learning. I think also that, in order to enhance the role of culture for stimulating tourism (as the authors recall in the title), they should devote more attention to the tourism sector. More theoretical explanations are necessary about the use of technologies in tourism experience, the main drivers and features of experience in tourism, and the role of culture in stimulating tourism. The link between tourism and the aim of the project (and the game) should be made clearer starting from the introduction section. In this section, authors could be more effective also avoiding confusion between the global and European level of analysis: they should focus to a wider and international level (UNESCO), and then to the Europe level, without mixing them.

 The empirical part of the study, devoted to the xFORMAL game, should better reflect the theoretical framework of the study, in order to allow to the readers to understand how the game is able to improve the informal education and to enhance the heritage tourism through the use of technology. More information on the game (where it has been tested, how the games and landscapes have been selected, what is the level of appreciation) could be useful to the readers.

 Conclusion should be better linked to the results that arises from the read of the game through the theoretical framework.

 At p. 7: at the end of the second line/second column, 18 and 19 have to be superscripts.

 The paper is well written and easy to understand.

Is the case presented with sufficient detail to be useful for teaching or other practitioners?

Yes

Is the work clearly and accurately presented and does it cite the current literature?

Partly

If applicable, is the statistical analysis and its interpretation appropriate?

Not applicable

Are all the source data underlying the results available to ensure full access and reuse by other researchers?

Partly

Are the conclusions drawn adequately supported by the data and analysis?

Yes

Is the background of the case’s history and progression described in sufficient detail?

Partly

Reviewer Expertise:

Tourism experience, smart destinations, cultural tourism

I confirm that I have read this submission and believe that I have an appropriate level of expertise to confirm that it is of an acceptable scientific standard, however I have significant reservations, as outlined above.
